# Differential and Interactive Effects of Substrate Topography and Chemistry on Human Mesenchymal Stem Cell Gene Expression

**DOI:** 10.3390/ijms19082344

**Published:** 2018-08-09

**Authors:** Qiongfang Li, Bo Zhang, Naresh Kasoju, Jinmin Ma, Aidong Yang, Zhanfeng Cui, Hui Wang, Hua Ye

**Affiliations:** 1 China National GeneBank-Shenzhen, BGI-Shenzhen, 518083 Shenzhen, China; liqiongfang@genomics.cn (Q.L.); majinmin@genomics.cn (J.M.); 2 Institute of Biomedical Engineering, Department of Engineering Science, University of Oxford, OX3 7DQ Oxford, UK; bo.zhang@eng.ox.ac.uk (B.Z.); naresh.kasoju@sctimst.ac.in (N.K.); zhanfeng.cui@eng.ox.ac.uk (Z.C.); 3 Department of Engineering Science, University of Oxford, OX1 3PJ Oxford, UK; aidong.yang@eng.ox.ac.uk; 4 Oxford Suzhou Centre for Advanced Research, Suzhou Industrial Park, 215123 Suzhou, China

**Keywords:** human mesenchymal stromal cells, cell matrix interactions, electrospinning, next generation sequencing, transcriptome

## Abstract

Variations in substrate chemistry and the micro-structure were shown to have a significant effect on the biology of human mesenchymal stromal cells (hMSCs). This occurs when differences in the surface properties indirectly modulate pathways within numerous signaling networks that control cell fate. To understand how the surface features affect hMSC gene expression, we performed RNA-sequencing analysis of bone marrow-derived hMSCs cultured on tissue culture-treated polystyrene (TCP) and poly(l-lactide) (PLLA) based substrates of differing topography (Fl: flat and Fs: fibrous) and chemistry (Pr: pristine and Am: aminated). Whilst 80% of gene expression remained similar for cells cultured on test substrates, the analysis of differentially expressed genes (DEGs) revealed that surface topography significantly altered gene expression more than surface chemistry. The Fl and Fs topologies introduced opposite directional alternations in gene expression when compared to TCP control. In addition, the effect of chemical treatment interacted with that of topography in a synergistic manner with the Pr samples promoting more DEGs than Am samples in all gene ontology function groups. These findings not only highlight the significance of the culture surface on regulating the overall gene expression profile but also provide novel insights into cell-material interactions that could help further design the next-generation biomaterials to facilitate hMSC applications. At the same time, further studies are required to investigate whether or not the observations noted correlate with subsequent protein expression and functionality of cells.

## 1. Introduction

Human mesenchymal stromal cells (hMSCs) are multi-potent, plastic-adherent, and fibroblast-like cells found in the human body. They were first discovered in bone marrow, but, subsequently, various non-marrow tissues were found to harbor similar cells [1,2,3,4]. In terms of potency, hMSCs of a particular tissue type were found to differentiate into cell types of tissue-specific lineage. However, with the advent of more developed in vitro culture protocols, it is now possible to achieve a cross-lineage differentiation [5,6]. Besides this intrinsic multi-potent property, several other beneficial characteristics of MSCs have recently been discovered. For instance, they have been found to facilitate neovascularisation both in the context of pathology and regeneration [7,8], the modulation of the immune response both innate and adaptive [9,10], and protection of certain cells by compromising auto-immunity, cytotoxicity, or the inflammatory response [11,12]. hMSCs are becoming an attractive source of cells for application in regenerative medicine. However, translation from bench to bedside requires better understanding of how environmental factors affect cells during cell isolation, in vitro expansion, and/or differentiation and delivery. Conventional approaches such as optimizing glucose, oxygen, and other culture conditions to maintain the expansion rate and the use of novel biochemical cocktails to control the differentiation are promising for enabling large-scale production of therapeutic stem cells.

More recently, biomaterials are being explored in various aspects of the research involving MSCs [13,14,15,16]. It is crucial for understanding cell-biomaterial interactions in order to maximize the potential in the designed material since many cellular processes including adhesion, migration, and stem cell differentiation are governed by the non-soluble components of the matrix. To this end, enhanced cell biological activities and narrowed integrin usage of cells cultured on three-dimensional matrices compared to their two-dimensional counterparts was shown to highlight the importance of substrate topography in regulating cell behavior and paracrine functions [17,18]. Additionally, various studies have shown enhanced osteogenic capacities with three-dimensional fibrous culture surfaces [19,20,21,22,23] and have shown regulations of the differentiation potential of other lineages [24,25,26,27]. Additionally, the induction of osteogenic and adipogenic differentiation on amine functionalized surfaces in contrast to pristine samples showed the effect of surface chemistry on the cell response [28]. Despite the various studies on understanding the role of the culture substrate topography and chemistry in modulating cell behavior, the detailed mechanism and impact remain to be elucidated. This is due to the limitations associated with conventional cellular scale assays such as microscopy and molecular scale assays such as qRTPCR. Given the complex interplay of several signaling networks that control the MSCs fate [29,30,31,32,33,34,35], landscape-scale understanding of the MSCs molecular response is needed to capture a detailed, global representation in a change in cellular status.

**Figure 1:**
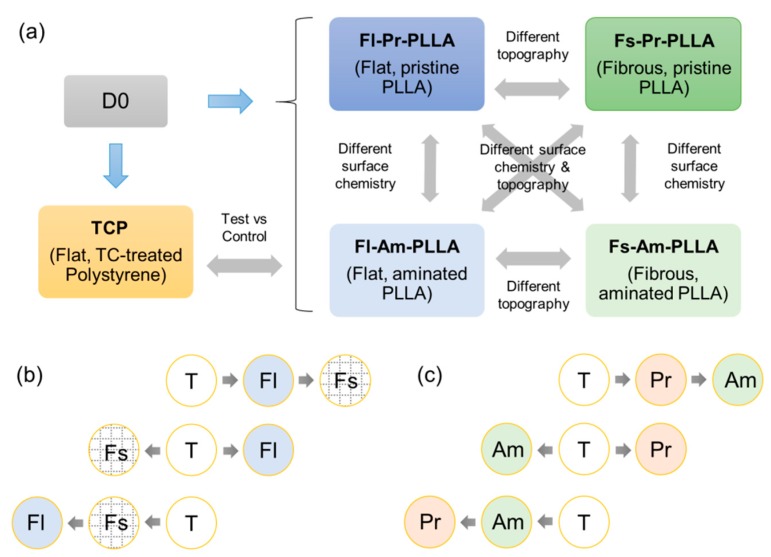
Schematics of potential relationships and underlying parameters: Experimental design and underlying variability within the test samples in terms of substrate topography and chemistry. (**a**) Hypothesized relationships between MSCs cultured on test substrates that differ in surface topography (**b**) and surface chemistry (**c**). In the latter case (b and c), the arrow direction indicates the gene expression characteristics between samples in relation to T (cells cultured on standard tissue culture dish).Hypothesis 1 (top) shows a gradual effect where Fs dominates Fl and Am dominates Pr, hypothesis 2 (middle) shows a directional polarity effect, and hypothesis 3 (bottom) shows another gradual change where Fl dominates Fs and Pr dominates Am. Starting cell suspension (before cell seeding) was assayed at day 0 (D0) while the cells grown on the test and the control samples were assayed at days 3, 5, and 7.

In this regard, high-throughput assays such as microarray and next generation sequencing-based transcriptomics have advanced the field of stem cell biology and technology [36]. Particularly, whole transcriptome shotgun sequencing known as RNA-sequencing or RNA-seq for short, is currently being explored in order to understand the gene expression profile of MSCs in various contexts [36]. One of the areas of significant interest is in the expansion of MSCs. Currently, flat or nanofibrous substrates-based surfaces are being actively investigated in cell cultures [24,37]. However, knowledge is limited regarding the molecular differences induced by surface topography or by changes in surface material from the tissue culture treated polystyrene substrate (TCP). The effect of culture surfaces on regulating MSCs differentiation, proliferation, paracrine function, and stemness preservation have been reported by various studies [23,38,39]. To design the culture surfaces with the topography and chemistry that are best suited for different downstream applications such as in vitro expansion and transplantation to facilitate tissue repair, it is important to gain an overall understanding of the induced gene expression changes. The fundamental questions that need to be addressed are (i) whether surface topography and chemistry mediate gradual changes or directional alternations and (ii) what kind of interactive effects they would impose on each other (Figure 1). To answer these questions, in the current study, we performed RNA-seq analysis on human bone marrow-derived MSCs (hbm-MSCs) cultured on flat (Fl) and fibrous (Fs) poly(L-lactide) (PLLA) substrates. Variables of surface chemistry were also introduced by modifying the PLLA substrates by using aminolysis (Am: aminated) in comparison to untreated substrates (Pr: pristine). The relative variations and the relationships between hMSCs cultured on Fl-Pr-PLLA, Fl-Am-PLLA, Fs-Pr-PLLA, Fs-Am-PLLA (test samples), and TCP (control sample) and the analytical strategy for investigating the interactions between topography and chemistry are shown in Figure 1.

## 2. Results and Discussion

### 2.1. Characteristics of Material

**Figure 2:**
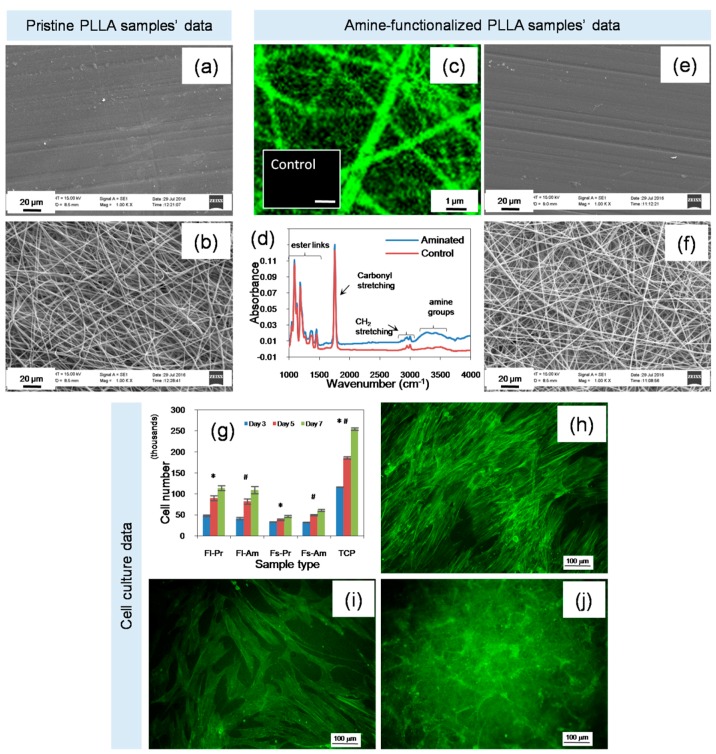
Substrate characteristics and cell response: SEM analysis showed the flat surface of Fl-Pr-PLLA (**a**) and the fibrous surface of Fs-Pr-PLLA. (**b**) FITC staining of Fs-Am-PLLA compared to Fs-Pr-PLLA (control) demonstrated the introduction of amine groups. (**c**) ATR-FTIR analysis of Fs-Am-PLLA further confirmed the successful amination against the untreated control without detrimental changes in the bulk structure. (**d**) SEM analysis of functionalized samples revealed the intactness of the morphological properties. (**e**,**f**) The Alamar blue assay suggested a higher cell proliferation on TCP followed by Fl-PLLA and then Fs-PLLA. (**g**) Statistically significant differences between groups compared are denoted by * or #, *t-*test *p* < 0.001, respectively). Lastly, confocal microscopy of cells stained for cytoskeleton with Alexa Fluor^®^ 488 phalloidin revealed relative differences in cell morphology and numbers (**h**) TCP, (**i**) Fl-PLLA, and (**j**) Fs-PLLA.

In this study, we have chosen a commonly used biocompatible polymer, PLLA, processed it into either a flat or fibrous material, and compared the results of the cell culture with cultures on standard TCP. Furthermore, the PLLA substrates were subjected to aminolysis to incorporate amine groups and, thereby, to alter the surface chemistry from hydrophobic to hydrophilic [40]. The morphological features, which were analyzed by SEM, are presented in Figure 2a,b. The flat substrate was made by melt compression molding and the fibrous substrate was made by electrospinning with a mean fiber diameter of 603 nm (± 197 nm SD). The surface functionalization was confirmed by using a FITC staining technique where upon specific binding to amine groups, light emission at 515 nm is increased when excited at 492 nm (Figure 2c). In ATR-FTIR spectroscopy, the aminated substrate showed characteristic peaks with a significant peak at 3300 cm^−1^ and two insignificant peaks at 1650 and 1550 cm^−1^ [41,42]. This confirmed the success of the amination reaction while the core structure of the polymer appears to remain unaffected (Figure 2d). Furthermore, as evident from SEM imaging, the surface morphology was not altered after the functionalization reaction (Figure 2e,f).

### 2.2. Cell Growth and Morphology

The conventional cellular assays such as the Alamar blue-based cell viability test and the microscopy-based cell morphology analysis are simple yet invaluable tools to obtain first-hand information on the cell response. Analysis using a cell metabolic assay (Alamar Blue) revealed that MSCs seeded on standard TCP proliferated more rapidly than those cultured on the test materials (*p*< 0.001, *t*-test, Figure 2g). Cytoskeleton imaging also confirmed that the cell number and cellular spreading were relatively higher on TCP than on the test materials and cells on flat PLLA film showed more similar morphology to those on TCP (Figure 2h–j). These observations suggest that the hbm-MSCs favored a flat surface. The overall trend in the cellular rate of proliferation was the highest on TCP followed by on Fl-PLLA and then on Fs-PLLA. Among the test conditions, topography made a significant difference (F = 39.27, df = 1, *p* < 0.001, GLM) on cell proliferation while chemistry was not a significant factor (F = 0.03, df = 1, *p* = 0.868, GLM). There was no significant interaction (F = 1.56, df = 1, *p* = 0.217, GLM) between topography and chemistry based on the cell proliferation data. This behavior may be caused by a lack of RGD (Arg-Gly-Asp) or other cell adhesion molecules on the PLLA substrates, the overwhelming effect of serum presence in the culture medium over the modification on surface chemistry, or the low sensitivity of the applied amine group in affecting the cellular proliferation assay [43,44,45,46]. Recent studies showed that the substrate properties including topography and composition as well as culture conditions significantly influence the bm-MSC response including cell proliferation, colony forming efficiency, tri-lineage differentiation potential, and immunomodulatory ability [47,48].

### 2.3. RNA-Seq Data Analysis

For the day 3 hbm-MSCs in this study, over 134 million clean reads were reported with a minimum quality score (Q20) of 89.7%. Over 69% of reads were identified as uniquely mapped human genes. Sequencing saturation data confirmed that a sufficient number of genes have been identified for all six samples (Supplementary Figure S1). The distribution of reads across a full gene length showed an acceptable level of sequencing randomness (Supplementary Figure S2).

**Figure 3:**
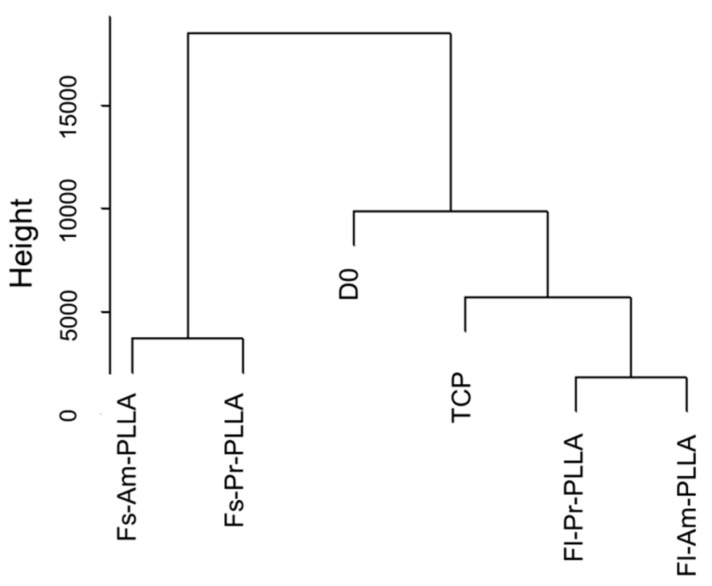
Unsupervised hierarchical clustering dendrogram: Relative sample similarities are represented by the distance along the *y*-axis. Samples or sample groups with greater similarities are arranged under the same hierarchy clades.

The expression levels of all detected genes were calculated as fragments per kilobase of transcript per million mapped reads (FPKM) (Supplementary Table S1). The results of unsupervised clustering on the six samples using all detected genes are shown in Figure 3. The largest Euclidean distance was displayed between the topography treatments Fl-PLLA and Fs-PLLA while close similarities were detected between chemistry treatments, i.e., Fl-Pr-PLLA to Fl-Am-PLLA and Fs-Pr-PLLA to Fs-Am-PLLA. Such a difference in distance clearly indicated that the surface topography was a dominating factor over the surface chemistry for inducing gene expression changes in hbm-MSCs. It was surprising that the two control samples (D0 and TCP) did not exhibit the largest differences from either of the two topography treatments (Figure 3), which supports the polarity model of topography postulated in Figure 1b (middle row). When TCP and PLLA samples were compared, TCP showed a closer resemblance to Fl-PLLA samples than to Fs-PLLA samples, which indicated that the Fs substrate was more efficient to alter hbm-MSCs gene expression than Fl substrate. To our knowledge, both the topography polarity model and the difference of Fs and Fl efficiency on altering hbm-MSCs gene expression have not been previously reported in the literature.

**Figure 4:**
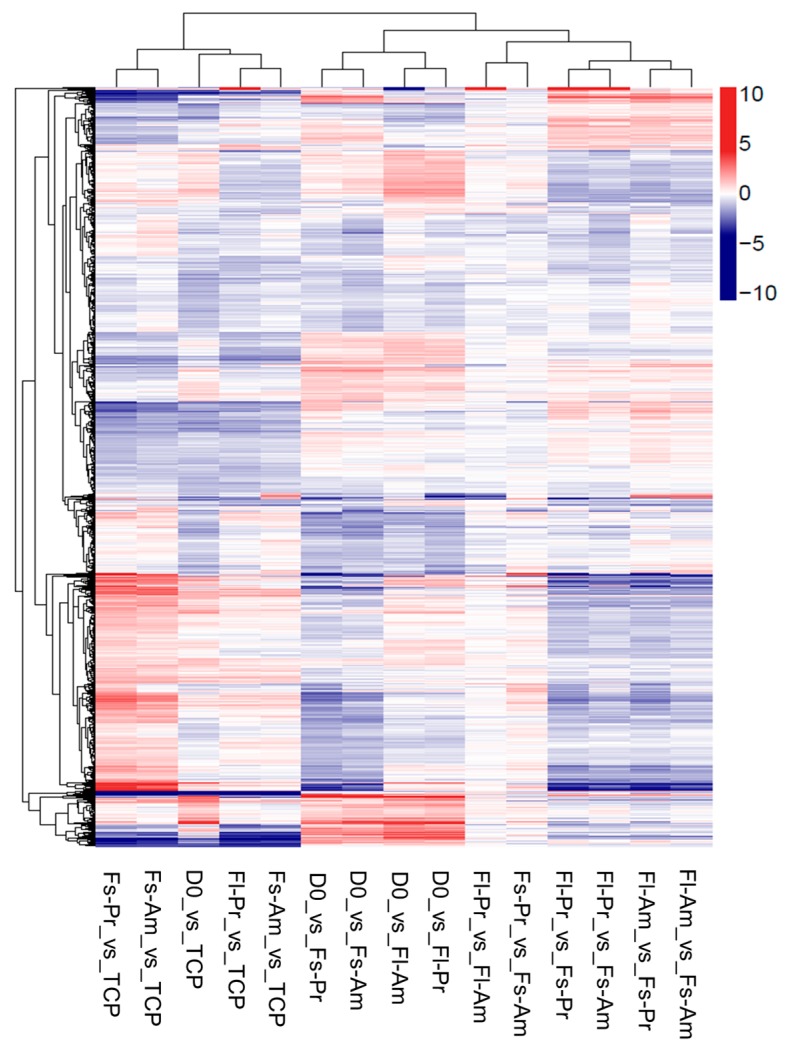
Comparison between DEGs from each sample pairs, which includes hierarchical cluster analysis of the DEGs between each sample pair. The sample pairs are listed on the primary *x*-axis while all reported DEGs are listed on the *y*-axis with their expression ratio (log_2_) expressed by the color gradient shown. The secondary *x*-axis groups the results based on the similarity in the sample pairs. Gene clustering is presented on the secondary *y*-axis.

Hierarchical cluster analysis was also used to represent pairwise FPKM comparisons for detecting DEGs (Figure 4). As shown by two columns with the least color gradient, amination treatment (Fl-Pr vs. Fl-Am and Fs-Pr vs. Fs-Am) induced relatively small numbers of DEGs. The clustering results on DEGs confirmed earlier findings (Figure 3), i.e., the effect of surface chemistry is less significant than that of surface topography and the gene expression of TCP shows greater similarity to Fl-PLLA than to Fs-PLLA samples. The change in gene expression levels between Fl-PLLA, Fs-PLLA, and TCP is greater. However, the ensemble expression results again supported the polarity model (directional alternation) in Figure 1b.

**Figure 5:**
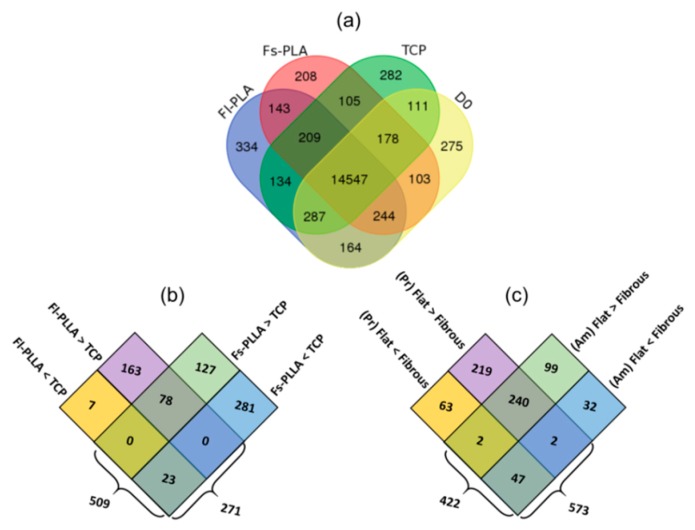
The sequencing summary and comparative analysis of samples cultured on different surfaces. (**a**) Venn diagram indicating the number of common genes detected for different samples. Common genes between Fl-PLLA and Fs-PLLA samples with Am and Pr functionalization treatments are extracted and compared with TCP and D0 samples. (**b**) The number of DEGs in Fl-PLLA and Fs-PLLA when compared to the TCP sample. (**c**) The number of DEGs between Pr-PLLA and Am-PLLA samples. In all cases, unique or shared up-regulated genes in each sample are shown.

A total of 14,547 non-differentially expressed genes were detected among all six samples (Figure 5a). This group contains certain housekeeping genes that are responsible for basic cellular functions, metabolism, cytokines, stem cell characteristic genes, and hMSCs markers. In total, 277 gene expressions were either detected uniquely for a particular sample or shown to be differentially up-regulated or down-regulated by different culture conditions. Such a differential response of MSCs, in terms of proliferative and differentiation potential, towards surface topography or surface chemistry have been reported in the literature [25,49,50,51]. Taking the advantages of transcriptome sequencing, DEGs were sorted to eight sets to further understand the topography (Figure 5b) and chemistry impact (Figure 5c) on altering hbm-MSCs gene expression patterns.

Earlier findings have shown that surface topography was more effective than chemistry at inducing hbm-MSCs gene expression changes (Figure 3 and Figure 4) and that the topography treatments fit with the polarity model (Figure 1b). The two topography sample sets (including both chemistry treatments Pr-PLLA and Am-PLLA) were compared respectively to the TCP control. A higher number of DEGs between Fs-PLLA and TCP than that of Fl-PLLA and TCP (Figure 5b) confirmed the findings in Figure 3, which showed a greater differential distance between the two groups. A total of 271 DEGs were found between Fl-PLLA and TCP among which 241 genes (89%) were up-regulated in Fl-PLLA. Contrastingly, among the 509 DEGs between Fs-PLLA and TCP, 304 genes (60%) were down-regulated in Fs-PLLA. There existed 78 and 23 common genes that were up-regulated and down-regulated, respectively, in both Fl-PLLA and Fs-PLLA when compared to TCP (Figure 5b). Detection of these shared DEGs suggested that Fl and Fs induced gene expression changes that were not completely opposite even though the majority of the DEGs were unique to either Fl or Fs. Fl uniquely induced 163 up-regulations and only one down-regulation to TCP while Fs uniquely induced 127 up-regulations and 281 down-regulations (Figure 5b). The significant difference (χ^2^ = 218.038, df = 1, *p* < 0.001) of up-regulated DEGs and down-regulated DEGs between Fl and Fs further supported the polarity model (Figure 1b).

**Figure 6:**
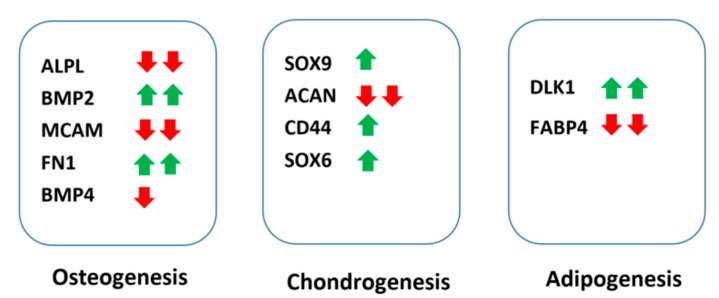
Differences in expression of key differentiation markers: in comparison to cells cultured on flat surfaces, cells cultured on fibrous matrices exhibited significant up-regulation (up arrow) and down-regulation (down arrow) in osteogenic, chondrogenic, and adipogenic markers. One arrow labels fold changes < 1.75 and two arrows label fold changes > 1.75.

Key differentiation markers were significantly altered by culturing with fibrous surfaces when comparing the Fs and Fl samples (Figure 6). Among osteogenic markers, ALPL, MCAM, and BMP4 were down-regulated and BMP2 and FN1 were up-regulated in Fs while RUNX2 remained unaltered (Table S2). ACAN, which is one of the chondrogenic markers, was down-regulated in Fs while SOX9, CD44, and SOX6 displayed notable increases. The expression levels for the adipogenic marker, FABP4, were down-regulated and a negative regular, DLK1, was up-regulated in Fs. Comparing to the markers for osteogenesis and chondrogenesis, the adipogenic markers have shown consistently low expression in all samples. Expression levels of all differentiation markers are included in Table S2. However, the recorded findings were indicative of the early fate decisions in hbm-MSCs caused by the substrate properties. Furthermore, the cells from the Fs samples are not believed to be constrained by the nutrient diffusion of the microenvironment. The local shortage of dissolved oxygen would upregulate expression levels of certain hypoxic proteins, e.g., MMP9, VEGF, MIF, PGK1, HIF1A, LDHA, TWIST, HGF, FGF, etc. [52,53,54,55,56,57]. Yet, none of these proteins were found to be upregulated in the Fs samples. The thickness of the synthesized fibrous surface was in the same magnitude of the peripheral zone (100–300 μm) reported in the literature [58,59,60].

To examine the chemistry-topography interaction, i.e., the impact of the chemistry factor on topography induced gene expression changes, DEGs of Fl-Pr-PLLA vs Fs-Pr-PLLA were compared with DEGs of Fl-Am-PLLA vs Fs-Am-PLLA (Figure 5c). Separate from Figure 5b, there were 291 shared DEGs between the two chemistry treatments while 282 DEGs were unique for Pr-PLLA and 131 DEGs were unique for Am-PLLA. Among the 291 shared DEGs, 240 and 47 common genes were found respectively to be up-regulated and down-regulated in the comparison between Fl-PLLA and Fs-PLLA samples, which were insensitive to the difference in chemistry, while only four DEGs showed opposite trends between samples with the two chemistry conditions. These DEGs with the similar pattern in both sets of comparisons (Figure 5c) represented the dominant responses in the chemistry-topography interaction and may further suggest a synergistic rule between chemistry and topography. Such a synergistic effect between surface topography and chemistry was reported earlier by Li et al. in regulating mesenchymal markers in cancer cells. However, a different set of modifications on topography and surface chemistry was used in their study. Therefore, the level of synergistic effects may depend on different combinations of abiotic culture conditions [61].

**Figure 7:**
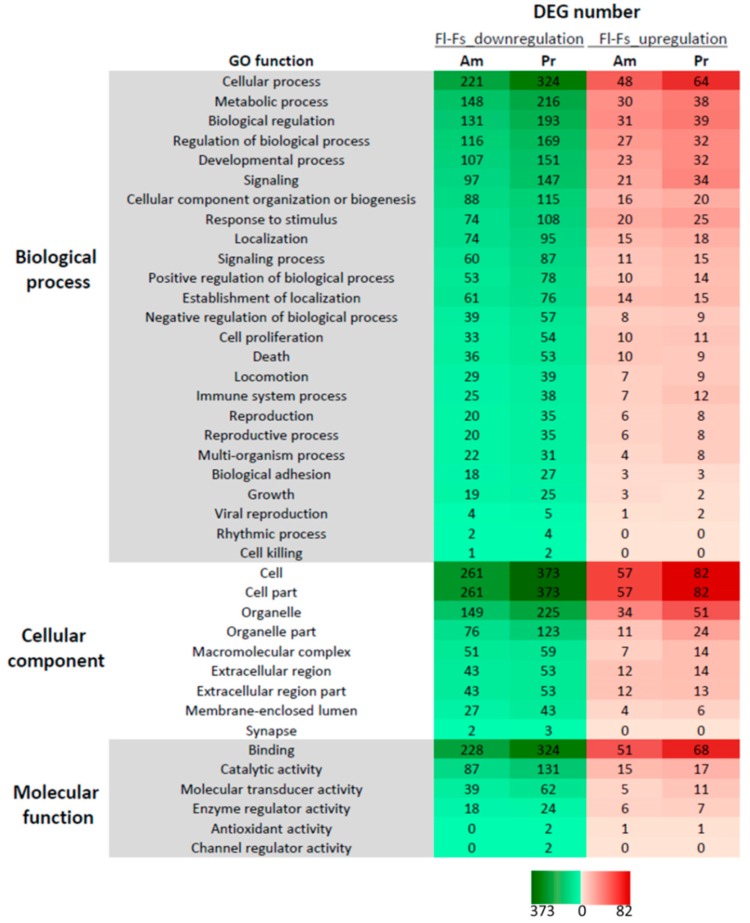
Gene ontology analysis for DEGs: DEGs associated with biological processes, cellular components, and molecular function were grouped and counted.

In spite of the supporting evidence on the polarity model of the topography factor (Figure 1b, middle row) between Pr-PLLA and Am-PLLA, the numbers of unique up-regulations and down-regulations (Figure 5c) were not statistically different (χ^2^ = 0.220, df = 1, *p* = 0.639) against the polarity model but did support the gradual change models of the chemistry factor. In this regard, the chemistry model T-Am-Pr (Figure 1c, bottom row) was supported, which indicates that Pr-PLLA was synergistically more powerful than Am-PLLA to induce hbm-MSCs gene expression alternations in response to topography treatments. To further clarify the interactions between chemistry and topography treatments, changes in the key differentiation markers and gene ontology (GO) analysis was performed by comparing the Fl-PLLA and Fs-PLLA samples. The numbers of DEGs associated with GO functions were shown in Figure 7. The proportion of genes corresponding to each function remained similar for Pr-PLLA and Am-PLLA. The majority of the DEGs were associated with cellular biogenesis, metabolism, binding, and organelle functions. In all of the detected GO functions, Pr-PLLA induced more DEGs than Am-PLLA, which highlights that Pr-PLLA made hbm-MSCs more sensitive with regard to responding to the topography treatments than Am-PLLA did, which provides further evidence to support the gradual model of T-Am-Pr for surface chemistry (Figure 1c, bottom row).

The scope of this study focuses on the impact of culture surfaces on the transcriptome profile of hbm- MSCs. The GO results shed light on the commonly associated functionality of the genes. However, the variation observed at the gene level may not be representative for the corresponding proteomic profile and the subsequent functionality of the cells. Future work will focus on verifying whether the changes observed in gene expressions resemble the alteration on cell functions and their corresponding protein expressions.

## 3. Materials and Methods

### 3.1. Materials

Purasorb PL 24 (PLLA with an inherent viscosity of 2.38 dL/g) was purchased from Corbion, Amsterdam, Netherlands. Other reagents used in the material preparation were, unless otherwise stated, purchased from Sigma-Aldrich, St. Louis, MO, USA. Other cell culture related reagents were, unless otherwise stated, purchased from Thermo Fisher Scientific Inc, Waltham, MA, USA. The RNeasy Mini kit was purchased from Qiagen, Hilden, Germany.

### 3.2. Study Groups

D0: cell suspension before seeding onto the test and control substrates. TCPS are cells cultured on tissue culture treated polystyrene dishes. Fl-Pr-PLLA are cells cultured on flat pristine PLLA electrospun mats. Fl-Am-PLLA are cells cultured on flat aminated PLLA electrospun mats. Fs-Pr-PLLA are cells cultured on fibrous pristine PLLA electrospun mats and Fs-Am-PLLA are cells cultured on fibrous aminated PLLA electrospun mats.

### 3.3. Preparation and Characterization of PLLA Substrates

Flat films of PLLA (Fl-PLLA) were prepared by the melt-pressing technique. The PLLA granules were placed between aluminum foil-covered metal plates and were pressed using a portable hot press at 210 °C for 15 min. The hot metal plates were carefully snap-cooled under running water. The PLLA film was then recovered from the plates. The nonwoven fibrous matrices of PLLA (Fs-PLLA) were prepared by the electrospinning method. The PLLA granules were dissolved in hexafluoroisopropanol to make a 7.5% (*w*/*v*) solution and the electrospinning was done in a custom-made setup at following optimized parameters: the voltage of 15 kV, which is a distance of 15 cm distance, and the flow rate of 0.1 mL/h.

To introduce the amine groups and to turn the surface from hydrophobic to hydrophilic, the PLLA substrates, which are both flat and fibrous, were subjected to aminolysis or amination reaction. First, they were saturated in 5 ml of isopropanol for 30 min at ambient temperature. This step was carried under a slight vacuum to remove the trapped air especially from the electrospun scaffolds. The isopropanol was then exchanged with 5 ml of freshly prepared hexamethylenediamine (HMDA) solution (5% *w*/*v* in isopropanol) for 30 min at 30 °C under mild agitation. The samples were washed with an ample amount of ultrapure water for 4 h with at least 10× water exchanges to get rid of the excess HMDA.

To determine the surface morphology, the samples were initially coated with platinum for 90 s in a sputter coating unit (SC7620, Quorum Technologies, Lewes, UK) and were then analyzed by using a scanning electron microscope (SEM, Evo LS15, Carl Zeiss, Oberkochen, Germany). Next, to confirm the successful amination, the samples were stained with fluorescein isothiocyanate (FITC, 0.05% *w*/*v* in absolute ethanol) for 4 h followed by imaging with a confocal laser scanning microscopy (CLSM, C2^+^, Nikon; excitation wavelength = 490 nm and emission wavelength = 525 nm). Any changes to substrate morphology and chemistry after aminolysis were tracked by SEM and attenuated total reflectance Fourier transform infrared spectroscopy (ATR-FTIR, Tensor 37, Bruker, Billerica, MA, USA).

### 3.4. Cell Culture

Poietics^™^ normal human bone marrow-derived mesenchymal stem cells (hbm-MSCs, cat. no. PT-2501) along with the proprietary growth medium kit (cat. no. PT-3001) were obtained from Lonza, UK. Cells were expanded in the proprietary hbm-MSCs growth medium supplemented with penicillin (10 U/mL) and streptomycin (10 µg/mL). Cells were cultured in 5% CO_2_ in air at 37 °C with a relative humidity of 95%. The PLLA substrates known as Fl-Pr-PLLA (flat, pristine PLLA), Fl-Am-PLLA (flat, aminated PLLA), Fs-Pr-PLLA (fibrous, pristine PLLA), and Fs-Am-PLLA (fibrous, aminated PLLA) were cut into 20 mm circular discs to fit into the wells of a 12-well plate. They were sterilized by treating with 70% ethanol for 1 h. A vacuum was applied to remove any trapped air especially in fibrous matrices. The samples were thoroughly washed with sterile phosphate buffered saline (PBS) and were then pre-saturated with culture medium overnight in a CO_2_ incubator. hbm-MSCs (passage 3) were seeded onto the substrate at a rate of 50,000 cells/well and cultured in the incubator for 24 h. Cells were cultured for one week with fresh media changes every alternative day. Cell viability at day 3, 5, and 7 was tested with an Alamar blue-based non-destructive assay. The 10% of Alamar blue reagent was added directly to each well. After 3 h of incubation, the supernatant was collected and the fluorescence was read in a multi-plate reader (SpectraMax M2, Molecular Devices, San Jose, CA, USA). Cell morphology was observed by staining the samples (paraformaldehyde fixed) with Alexa Fluor^®^ 488 phalloidin and the observation was conducted under a confocal microscope.

### 3.5. RNA Extraction

Total RNA from each sample was extracted with the RNeasy Mini Kit following supplier’s instructions. A total of 500 μL of lysis buffer was added to each sample and pipetted vigorously to rupture the cell membrane. An equal volume of 70% ethanol was added to allow the precipitation of nucleic acids. The mixture was then transferred to the RNeasy spin column to let the matrix capture the nucleic acids. The column with captured nucleic acids was washed with 500 μL of washing buffer and treated with 80 μL of DNase (10 μL DNase stock + 70 μL of buffer) to digest DNA. The digested DNA was then washed three times with 500 μL of washing buffer. Lastly, the RNA was eluted with 25 μL of RNase-free water and stored at –80 °C until further use. An aliquot of the extracted total RNA was analyzed for its quality and quantity by using a NanoDrop UV/VIS spectrophotometer (1000, Thermo Scientific, Waltham, MA, USA) and RNA agarose gel electrophoresis. It is to be noted that, for all the test samples including TCP, the lysis buffer was added directly to the cell culture well while, for the starting material (Figure 1a, Day 0 cell suspension), the lysis buffer was added to the cell suspension after trypsinization.

### 3.6. RNA-Seq and Data Analysis

Next generation sequencing was conducted on a BGISeq-500 sequencing platform (BGI, Shenzhen, China) for six samples (Day 0 cell suspension, Day 3 TCP control, and treatments of Fl-Pr-PLLA, Fl-Am-PLLA, Fs-Pr-PLLA, and Fs-Am-PLLA) with a read length of 100-bp. Raw sequencing data was filtered to remove low quality reads using SOAPnuke (http://soap.genomics.org.cn/) with the following criteria: reads with adapters, reads with greater than 10% unknown bases, or reads with more than 50% low-quality bases. The Bowtie2 [62] and HISAT [63] toolbox was used for gene and genome mapping against the human genome reference hg19, respectively. A unique gene and genome mapping percentage were calculated. Sequencing saturation and reads distribution checks were performed as quality controls to the alignment process. Gene expression levels for each sample were quantified using the FPKM method (fragments per kilobase of transcript per million mapped reads) and processed for analysis. Differentially expressed genes (DEGs) were determined in pairwise comparisons when the expression ratio was greater than 2 and the false discovery rate was less than 0.001. No upper or lower limit cut-off on the gene expression level was imposed to filter DEGs. The FPKM value was set to two decimal points to avoid error during downstream data processing. Hierarchical cluster analysis assessed the gene expression FPKM values between samples. Samples with fewer differences were grouped together with the result shown in a dendrogram. Gene ontology analysis classified and counted the number of genes responsible in the identified biological functions [64].

### 3.7. Statistical Analysis

For the cell culture measurements, all the studies were run in replicates of six. The qualitative data were illustrative of the individual group. The numerical data were presented as the Mean ± Standard Deviation (SD). Student *t-*tests were used to determine statistically significant differences of cell proliferation responses to topography and chemistry treatments. For RNA-seq, all replicates (*n* = 6) were pooled prior to sequencing. Differences on the number of up-regulated DEGs and down-regulated DEGs were determined by using the Chi-square test (*p* < 0.05).

## 4. Conclusions

While the effects of substrate topography and chemistry on hMSCs behavior are reported in the literature, we present a global perspective of the cell-material interactions. We explored RNA-seq to understand the differential gene expression in hMSCs cultured on TCP, Fl-Pr-PLLA, Fl-Am-PLLA, Fs-Pr-PLLA, and Fs-Am-PLLA. Various statistical analyses were performed to understand the gene expression patterns. The influence of surface topography (Fs-PLLA and Fl-PLLA samples) was much more influential on regulating gene expressions than the chemistry treatments (Am-PLLA and Pr-PLLA samples). On topography, the induced differences between TCP and Fs-PLLA samples were more polar than those between TCP and Fl-PLLA. Despite the relative small impact of the chemistry treatments, Pr-PLLA samples showed a higher sensitivity in strengthening the differentiating role of topography than the Am-PLLA samples. The tested independent factors of surface chemistry and topography imposed their effects on the overall gene expression profiles interactively. These findings offer an enriched understanding of the impact of culture surface properties on hMSCs gene expression and facilitate advanced planning when engineering in vitro culture materials for hMSCs. Meanwhile, further studies are needed to verify if the observations noted at a gene expression level correlates with subsequent protein expressions and functionality of cells.

## Supplementary Materials

Supplementary materials can be found at http://www.mdpi.com/1422-0067/19/8/2344/s1.

## Availability of data and materials

All the processed data are presented in the article. Information on raw data and materials are available from the NCBI BioProject number PRJNA395968 and from the corresponding authors upon request.
