# Correction: Li, Q., et al. Differential and Interactive Effects of Substrate Topography and Chemistry on Human Mesenchymal Stem Cell Gene Expression. *Int. J. Mol. Sci.* 2018, *19*, 2344

**DOI:** 10.3390/ijms20225652

**Published:** 2019-11-12

**Authors:** Qiongfang Li, Bo Zhang, Naresh Kasoju, Jinmin Ma, Aidong Yang, Zhanfeng Cui, Hui Wang, Hua Ye

**Affiliations:** 1 China National GeneBank-Shenzhen, BGI-Shenzhen, Shenzhen 518083, China; liqiongfang@genomics.cn (Q.L.); majinmin@genomics.cn (J.M.); 2 Institute of Biomedical Engineering, Department of Engineering Science, University of Oxford, Oxford OX3 7DQ, UK; bo.zhang@eng.ox.ac.uk (B.Z.); naresh.kasoju@sctimst.ac.in (N.K.); zhanfeng.cui@eng.ox.ac.uk (Z.C.); 3 Department of Engineering Science, University of Oxford, Oxford OX1 3PJ, UK; aidong.yang@eng.ox.ac.uk; 4 Oxford Suzhou Centre for Advanced Research, Suzhou Industrial Park, Suzhou 215123, China

The authors wish to make the following correction to this paper [1]: The authors regret the mislabeling of Figure 4. Lane-5 was mistakenly labeled as "Fs-Am_vs_TCP", the same as that of Lane-2. The correct label for Lane-5 should be "Fl-Am_vs_TCP". The mistake was generated during final editing following peer review. The mistake did not affect the review process. Our correction does not change the conclusions of this manuscript.

The corrected Figure 4 is shown below (Figure 1).

The authors would like to apologize for any inconvenience caused to the readers by these changes.

## Figures and Tables

**Figure 1:**
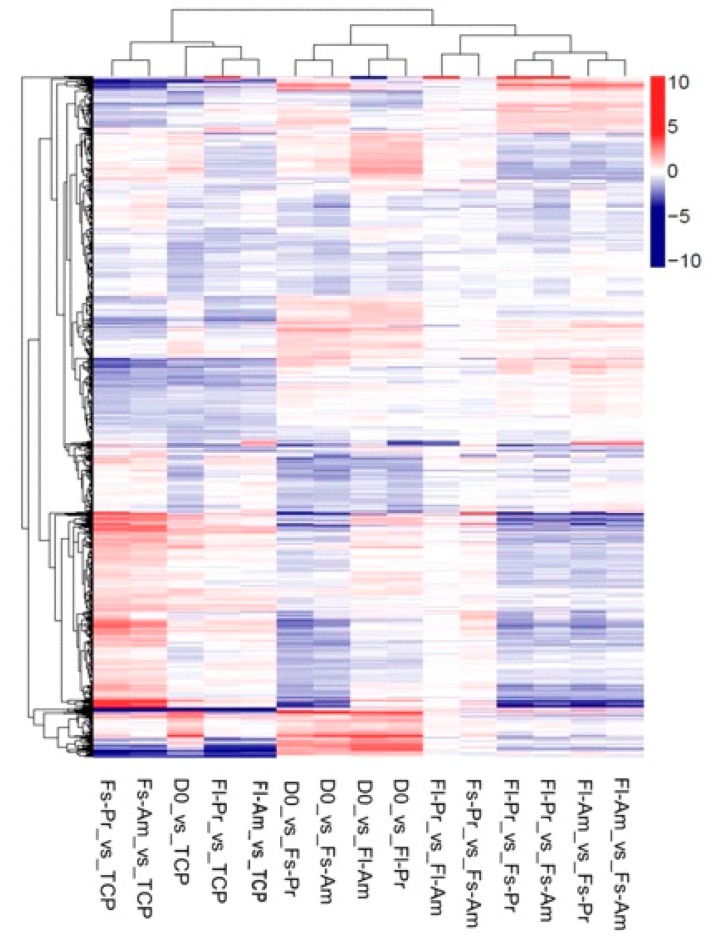
Between DEGs from each sample pair, which includes hierarchical cluster analysis of the DEGs between each sample pair. The sample pairs are listed on the primary *x*-axis, while all reported DEGs are listed on the *y*-axis with their expression ratio (log_2_) expressed by the color gradient shown. The secondary *x*-axis groups the results based on the similarity in the sample pairs. Gene clustering is presented on the secondary *y*-axis.
