# Supplementary material for: Differential and Interactive Effects of Substrate Topography and Chemistry on Human Mesenchymal Stem Cell Gene Expression
Source: Int J Mol Sci. 2018 Aug 9;19(8):2344. doi: 10.3390/ijms19082344 (PMC6121573; doi:10.3390/ijms19082344)
Supplement: Supplementary file 1 [file ijms-19-02344-s001.zip › Li et al 2018_Figure_S1.pptx]

## Slide 1
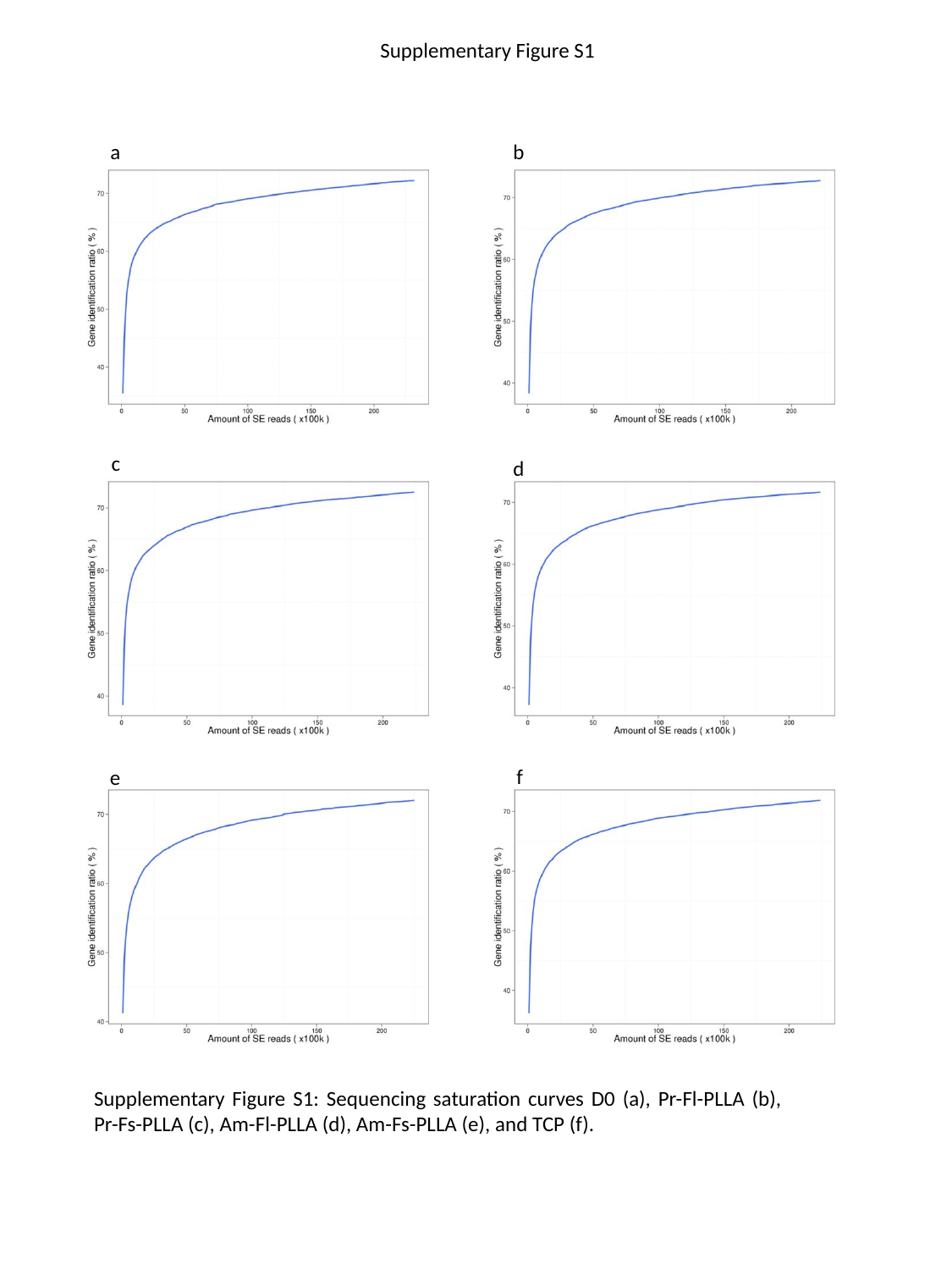

Supplementary Figure S1
a
b
c
d
f
e
Supplementary Figure S1: Sequencing saturation curves D0 (a), Pr-Fl-PLLA (b), Pr-Fs-PLLA (c), Am-Fl-PLLA (d), Am-Fs-PLLA (e), and TCP (f).
